# Real-World Specialty-Based Differences in the Diagnosis and Management of Tardive Dyskinesia: Results of a Physician Survey From Italy, Spain, France, and the United Kingdom

**DOI:** 10.1192/j.eurpsy.2026.10727

**Published:** 2026-07-31

**Authors:** A. Fagiolini, M. T. Driessen, A. Guevara Morel, M. Konings, K. Duma, S. Reed, P. Le Calvé, A. Sadou, S. Pappa

**Affiliations:** 1 University of Siena, Department of Molecular and Developmental Medicine, Siena, Italy; 2 Teva Pharmaceuticals; 3 Teva Pharmaceuticals Europe B.V, Haarlem, Netherlands; 4 Teva Branded Pharmaceutical Products R&D LLC, Parsippany, NJ, United States; 5 Teva Pharmaceuticals EU, Amsterdam, Netherlands; 6 Oracle Life Sciences, Paris, France; 7 Imperial College London, Department of Brain Sciences, London, United Kingdom

## Abstract

**Introduction:**

Tardive dyskinesia (TD) is a persistent movement disorder associated with long-term antipsychotic (AP) use, often leading to physical, psychological, and social burden. In Europe, psychiatrists and movement disorder specialist neurologists (MDS neurologists) manage TD, but no formal treatment guidelines exist. Understanding specialty-specific differences in recognition and treatment is therefore important.

**Objectives:**

To understand standard-of-care approaches to TD between MDS neurologists and psychiatrists.

**Methods:**

Psychiatrists and MDS neurologists in Italy, Spain, France, and the UK with experience managing TD completed a 25‐minute web‐based survey. Results were analyzed descriptively.

**Results:**

334 physicians (241 psychiatrists, 93 MDS neurologists) completed the survey. MDS neurologists reported a higher proportion of patients with TD in their clinical practice than psychiatrists. To diagnose TD, similar proportions of psychiatrists (52%) and MDS neurologists (47%) reported frequently using DSM-5 criteria. Both specialties estimated that most patients had mild or moderate TD, though MDS neurologists reported a larger proportion of severe TD (21% vs 15%). TD severity was commonly reported as influencing TD management strategy (psychiatrists: 82%; MDS neurologists: 70%), followed by the impact of TD on quality of life (76% vs 65%). For severe TD, psychiatrists were more likely to modify, switch or discontinue APs (55% vs 50%), while MDS neurologists more often prescribed add-on treatments (44% vs 37%). Both specialties reported similar patterns for utilizing the “watch and wait” approach (mild TD: 29–30%; moderate TD: 13%; severe TD: 7–8%). Across TD severities, anticholinergics and benzodiazepines were more frequently prescribed as an add-on treatment by psychiatrists, while tetrabenazine (TBZ) and amantadine were more frequently prescribed by MDS neurologists. Both groups rated TBZ and other off-label therapies only modestly effective, safe, and satisfactory (mean scores 4–8/10), underscoring the limitations of available options.

**Conclusions:**

Psychiatrists more often modify AP treatment, while MDS neurologists favor pharmacological add-on therapies. Both prioritize TD severity in decision-making. These findings may help guide efforts to harmonize TD care across specialties and improve patient outcomes.

**Disclosure of Interest:**

A. Fagiolini Grant / Research support from: Angelini, Boehringer Ingelheim, Idorsia, Italfarmaco, Lundbeck, Janssen, Medicamenta, Mylan, Otsuka, Pfizer, Recordati, Rovi, Sunovion, Teva, and Viatris, Consultant of: Angelini, Boehringer Ingelheim, Idorsia, Italfarmaco, Lundbeck, Janssen, Medicamenta, Mylan, Otsuka, Pfizer, Recordati, Rovi, Sunovion, Teva, and Viatris, M. Driessen Shareholder of: Teva Pharmaceuticals, Employee of: Teva Pharmaceuticals, A. Guevara Morel Shareholder of: Teva Pharmaceuticals, Employee of: Teva Pharmaceuticals, M. Konings Shareholder of: Teva Pharmaceuticals, Employee of: Teva Pharmaceuticals, K. Duma Shareholder of: Teva Pharmaceuticals, Employee of: Teva Pharmaceuticals, S. Reed Employee of: Oracle Life Sciences, P. Le Calvé Employee of: Oracle Life Sciences, A. Sadou Employee of: Oracle Life Sciences, S. Pappa Grant / Research support from: Johnson & Johnson, Lundbeck, Otsuka, Recordati, Rovi, Sunovion, and Teva Pharmaceuticals, Consultant of: Johnson & Johnson, Lundbeck, Otsuka, Recordati, Rovi, Sunovion, and Teva Pharmaceuticals

